# A Thermoelectric MEMS Microwave Power Sensor with Inline Self-Detection Function

**DOI:** 10.3390/mi13020239

**Published:** 2022-01-31

**Authors:** Jiaqi Liu, Yang Hong, Jutao Wang, Chunhua Cai, Zhiqiang Zhang

**Affiliations:** 1 Key Laboratory of MEMS of the Ministry of Education, Southeast University, Nanjing 210096, China; 220191415@seu.edu.cn (J.L.); 220201613@seu.edu.cn (Y.H.); 2 Shanghai Key Laboratory of Multidimensional Information Processing, School of Communication and Electronic Engineering, East China Normal University, Shanghai 200062, China; 71215904065@stu.ecnu.edu.cn

**Keywords:** MEMS, microwave power sensor, self-detection, thermoelectric

## Abstract

In this paper, the design, fabrication and measurement of a thermoelectric MEMS microwave power sensor with the terminal load inline self-detection function is proposed. The structure of the sensor mainly includes a coplanar waveguide, a thermopile, two terminal load resistors and two calibration resistors. In order to realize the inline self-detection function, the load and calibration resistors are designed to form a voltage divider circuit. The fabrication of this sensor is compatible with the GaAs MMIC technology. The on-chip performance is tested by using a microwave experimental platform. The measured reflection loss is less than −10 dB at 0.1–10 GHz. When the bias voltage is not applied, the sensitivity of the sensor is 47.39 μV/mW@5 GHz and 32.58 μV/mW@10 GHz, respectively, and when the bias voltage is applied, the sensitivity is 47.50 μV/mW@5 GHz and 32.73 μV/mW@10 GHz, respectively. The difference between the two cases is less than 0.5% at the same frequency, which indicates that whether or not to apply the bias voltage has little effect on the sensitivity. In addition, when the calibration resistance is increased from 50 to 100 Ω, the current flowing through the load resistance is decreased under the same bias voltage. Therefore, the DC power consumed on the load resistance will be significantly reduced. This makes the measured and theoretical results show better agreement, thus verifying the validity of the design.

## 1. Introduction

Microwave power is one of the three important parameters that characterize the feature of microwave signals (amplitude, frequency and phase). It is essential for the microwave power measurement in the generation, transmission and reception of microwave signals. Power detection plays an important role in microwave and millimeter-wave wireless applications, such as modern personal communication systems and radar systems. However, these systems require that the power sensors have low volume, wide frequency band, low reflection loss, zero dc power consumption and compatibility with the GaAs monolithic microwave integrated circuit (MMIC) process, and so on [1,2,3].

The general method to detect microwave power measurement is employing termination power sensors such as diodes, thermistors or thermocouples. These traditional methods can meet the basic testing requirements, but they also show some shortcomings. For example, the diodes are an active device and non-linear under larger input power, the thermistors are temperature sensitive and low linearity, and the thermocouples have a large size and low sensitivity. Both of them run counter to the trend of high performance. Due to the increasing maturity of MEMS processing technology, the MEMS technology can be combined with the traditional microwave power measurement methods to produce more excellent MEMS microwave power sensors. The microwave power sensors based on the MEMS technology show advantages of low volume, wide frequency band, low reflection and insertion losses, zero dc power consumption, high sensitivity and linearity, compatibility with the GaAs MMIC process, etc. [4,5].

Recently, two typical kinds of microwave power sensors based on the MEMS technology have been widely studied. One, proposed by Dehe et al., is a thermoelectric-type MEMS microwave power sensor based on Seebeck effect. It excels in low reflection loss, good linearity and high sensitivity [6,7,8]. The other, proposed by Fernandez et al., is a capacitive-type MEMS microwave power sensor based on sensing the equivalent electrostatic force between the suspended beam and the microwave signal line [9,10,11]. It shows low reflection and insertion losses, high sensitivity and large dynamic range. In addition, these two types of power sensors have the characteristics of miniaturization, zero DC power consumption and low cost.

As one of the commonly used MEMS microwave power sensors, the thermoelectric power sensor based on the thermopile is provided with the characteristics of low loss, high linearity and wide band [12]. However, the impedance matching between the load resistors and the coplanar waveguide (CPW) is one of the key factors limiting the performance. It is found through investigations that this type of sensor embedded system has reliability problems such as aging or failure after long-term operation, which is usually manifested as an increase in the load resistance . Even the load resistance is able to burn out during high-power detection, causing the mismatch. In addition, with the rapid development of the Internet of Things, the transceiver components of RF terminal nodes are becoming more and more complex [13]. Therefore, in order to obtain convenient and accurate inline measurement of microwave power, the thermoelectric MEMS microwave power sensor with the terminal load inline self-detection function is proposed in this paper.

## 2. Structure and Design

**Figure 1:**
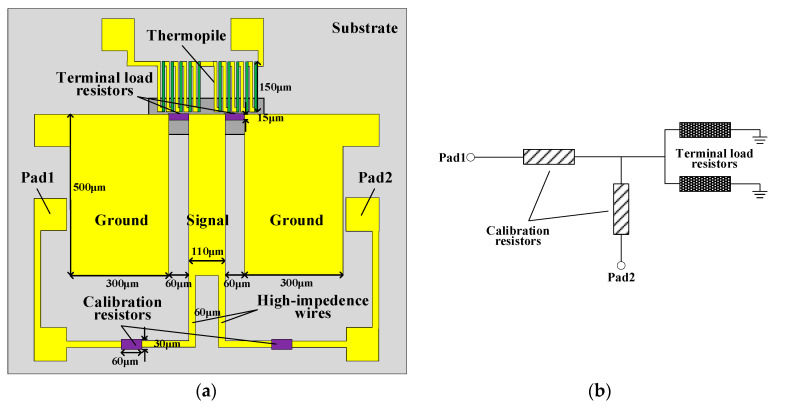
(**a**) The schematic diagram of the thermoelectric MEMS microwave power sensor with the inline self-detection function; (**b**) the simplified circuit diagram of the thermoelectric MEMS microwave power sensor with the inline self-detection function.

The structure of the thermoelectric MEMS microwave power sensor with the inline self-detection function is shown in Figure 1. The sensor is mainly composed of a CPW, two terminal load resistors, a thermopile, two calibration resistors and high-impedance wires. The CPW is used to transmit microwave power, and its characteristic impedance is designed to be 50 Ω calculated by ADS, and then optimized by HFSS software. The two load resistors are connected in parallel to the output of the CPW, where the length of the load resistor is determined by the spacing. To achieve matching with the CPW, each load resistor is designed to be 100 Ω. The thermopile consists of ten pairs of thermocouples in series and is placed near the load resistance, where the length is 150 μm. In order to increase the temperature difference between the hot and cold ends of the thermopile and thus improve sensitivity of the sensor, the length of the thermopile should be greater than the suspension length of the substrate membrane structure [14,15].

The operation principle of the MEMS microwave power sensors proposed in this manuscript is based on the conversion mechanism of microwave power–heat–electricity. The microwave power transmitted on the CPW is completely absorbed to generate heat by the two terminal load resistors. The thermopile is placed near the load resistors senses the surrounding temperature changes, resulting in a temperature difference between the two ends of the thermopile. The temperature difference is converted into a thermovoltage by the thermopile, based on the Seebeck effect. Thus, the sensors realize the measurement of microwave power by measuring DC voltage [16].

**Figure 2:**
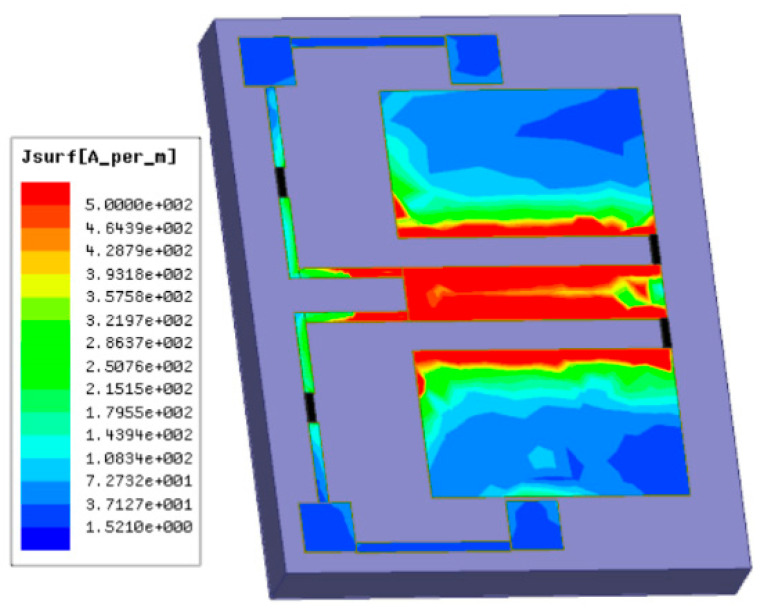
Current distribution of the thermoelectric MEMS microwave power sensor with the inline self-detection function.

In this sensor, the calibration resistors are connected to the signal line of the CPW through the high-impedance wires, respectively. The voltage divider circuit is formed by the calibration resistors and the terminal load resistors. The simplified circuit diagram of the thermoelectric MEMS microwave power sensor with the inline self-detection function is added and shown in Figure 1b. When a bias voltage is applied to pad 1, the partial voltage of the load resistors is measured on pad 2. Then the load resistors can be calculated while the sensor is working. The current distribution diagram of the thermoelectric MEMS microwave power sensor with the inline self-detection function is simulated by a HFSS software, as shown in Figure 2. The HFSS model uses lumped port excitation to simulate reflection loss parameters. The structural dimensions in Figure 1 are substituted into the simulation model. In the simulation setup, the CPW, pads and high-impedance wires are made of Au, the load and calibration resistors are made of TaN and the substrate material is GaAs. The simulated result shows the electromagnetic field of the thermoelectric MEMS microwave power sensor, and the effect of the high-impedance wires on the electromagnetic distribution of this power sensor.

## 3. Fabrication

**Figure 3:**
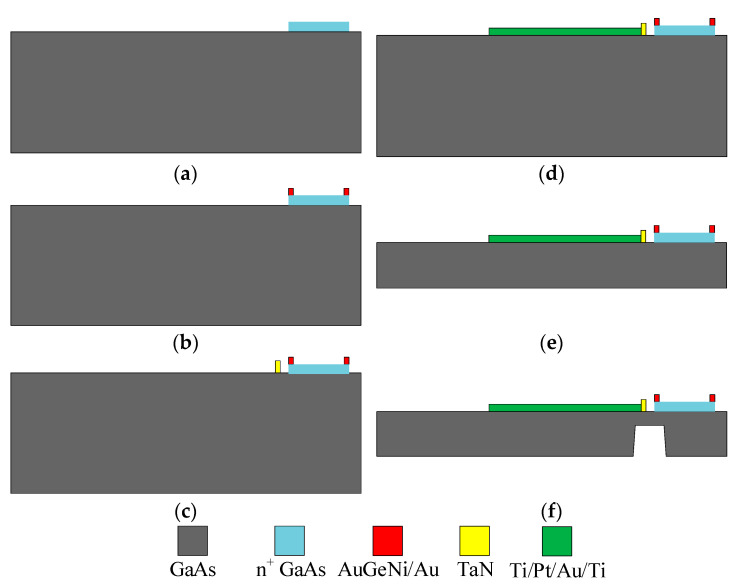
Process flow of the thermoelectric MEMS microwave power sensor with the inline self-detection function. (**a**) Etching n+ GaAs epitaxial layer; (**b**) sputtering AuGeNi/Au; (**c**) sputtering TaN; (**d**) evaporated Ti/Pt/Au/Ti; (**e**) thinning GaAs substrate; (**f**) forming a substrate film structure by the anisotropic dry-etching technology.

The thermoelectric MEMS microwave power sensor with the inline self-detection function is fabricated by using the GaAs MMIC process [17]. Figure 3 shows a specific process flow of this sensor. The corresponding specific process steps are as follows:

A GaAs substrate with the n+ GaAs epitaxial layer is chosen, where the thickness is about 625 μm. The ohmic contact areas and the semiconductor arms of the thermopile are formed through the photolithography and etching process; AuGeNi/Au is sputtered with a total thickness of 2700 Å, and the metal arms of the thermopile are formed by the lift-off process; TaN is sputtered with a sheet resistance of 25 Ω/Υ, and the terminal load resistors and the calibration resistors are formed by the lift-off process; Ti/Pt/Au/Ti is evaporated with a thickness of 500/300/3500/500 Å, and the CPW, the high-impedance wires and the pads are formed by the lift-off process; the GaAs substrate is thinned, and the thickness of the substrate is thinned to be 100 μm through the back grinding process; the GaAs substrate is etched underneath the terminal load resistors and the hot end of the thermopile by the anisotropic dry-etching technology to form a substrate film structure. The substrate with a thickness of about 90 μm is etched away, remaining a substrate film structure.

## 4. Testing and Discussion

**Figure 4:**
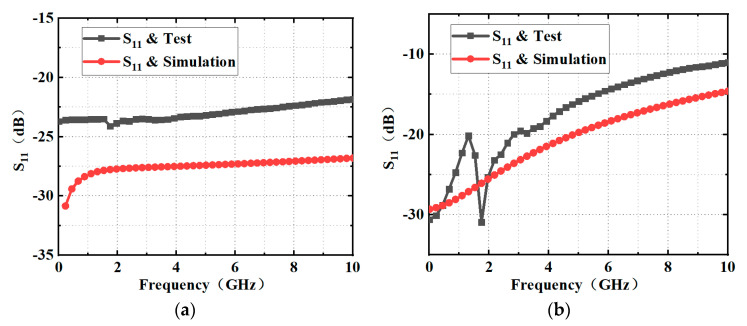
Simulated and tested reflection loss of the thermoelectric MEMS microwave power sensor (**a**) without the inline self-detection structure and (**b**) with the inline self-detection structure.

The reflection loss of the thermoelectric MEMS microwave power sensors with and without the inline self-detection structure is measured by using an Agilent N5224A network analyzer. In order to obtain an accurate measurement, the SOLT calibration method is adopted by the network analyzer. The simulated and tested reflection loss of the thermoelectric MEMS microwave power sensors with and without the inline self-detection structure is shown in Figure 4. The reflection loss of these two sensors is less than −10 dB at 0.1–10 GHz. Due to the introduction of the high-resistance wires and calibration resistors, the sensors with the self-detection structures have larger reflection loss. The maximum reflection loss is −11.15 dB at 10 GHz, but this only accounts for less than 10% of the total power. In addition, it can be observed through the comparison of the simulation and test curves that the tested reflection loss is slightly inferior. This is mainly due to the ideal settings of conductor and dielectric losses in the simulation.

**Figure 5:**
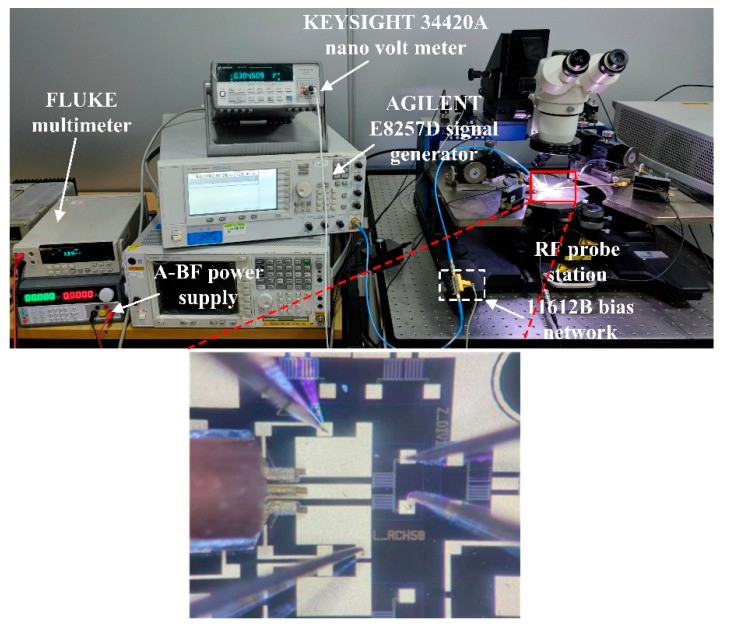
Photo of the test platform.

The sensing performance of these power sensors with and without the inline self-detection structure is tested by using a RF probe station, a 11612B bias network, a Agilent E8257D signal generator, a Keysight 34420A nano volt meter, a Fluke multimeter and an A-BF power supply. The photo of the test platform is shown in Figure 5.

**Figure 6:**
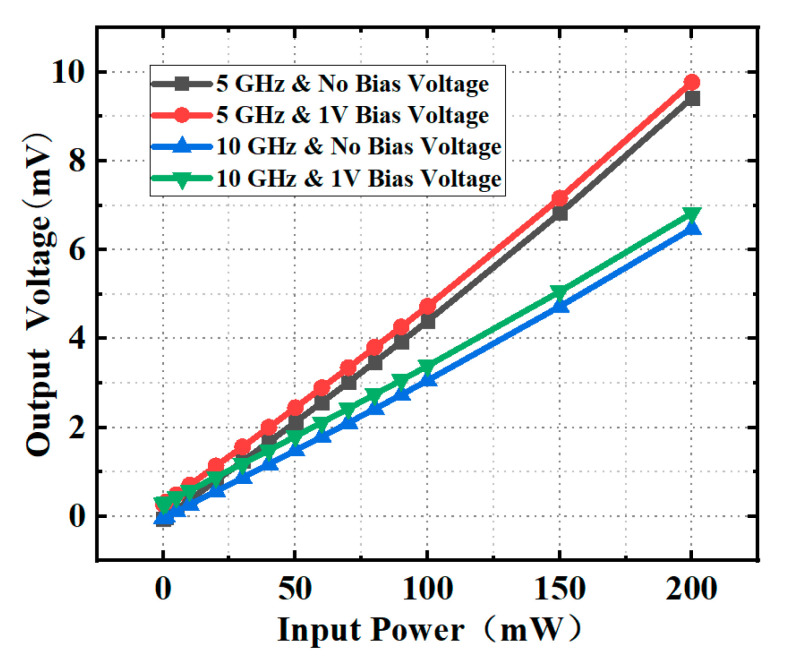
Tested relation between the input microwave power and the output voltage of the thermoelectric MEMS microwave power sensor with the self-detection function.

Figure 6 shows the tested relation between the input microwave power and the output voltage of the thermoelectric MEMS microwave power sensor with the self-detection function. In Figure 6, the output voltage of the sensor increases linearly with the input microwave power, and the sensitivity is 47.39 μV/mW@5 GHz and 32.58 μV/mW@10 GHz, respectively when the bias voltage is not applied. When the bias voltage is applied, the sensitivity of the sensor is 47.50 μV/mW@5 GHz and 32.73 μV/mW@10 GHz, respectively. The voltage difference is less than 0.5% at the same frequency, which indicates whether or not to apply a bias voltage has almost no effect on the sensitivity. Under applying the bias voltage, the additional DC power is consumed by the load resistors, which makes the output voltage increase for the same input microwave power. Therefore, there is a certain gap between the output voltages when the bias voltage is not applied and is applied, which is in the range of 310–380 μV. The load resistance can be detected inline at the same time as the microwave power measurement through the calibration method. The sensitivity of the thermoelectric-type MEMS power sensor is affected by the external temperature, but it shows a certain regularity. So the influence of temperature drift can be removed by means of the compensation. Some research work for the influence of temperature on the key performance of the sensor has been reported [18,19].

**Figure 7:**
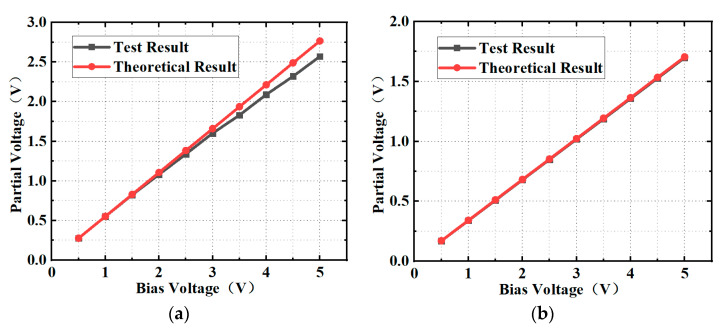
Relationship between the tested and theoretical results of the bias voltage versus the partial voltage on the load resistance for the calibration resistance of (**a**) 50 Ω and (**b**) 100 Ω.

The relation between the tested and theoretical results of the bias voltage versus the partial voltage on the load resistance for different calibration resistance is shown in Figure 7. It can be seen from Figure 7a that for the calibration resistance of 50 Ω, the tested result is slightly lower than the theoretical result, and the gap between them gradually increases as the bias voltage increases. This is because the DC power consumed on the load resistance increases, which causes the temperature of the load resistors with a negative temperature coefficient to increase, and the corresponding resistance decreases.

As for the calibration resistance of 100 Ω in Figure 7b, the current of the load resistors is reduced for the same bias voltage. That is, the DC power consumed on the load resistors is significantly reduced. It shows that the test and theoretical results show better agreement, thereby verifying the validity of the design.

## 5. Conclusions

This paper proposes the thermoelectric MEMS microwave power sensor with the inline self-detection function, which is based on the principle of microwave power–heat–electricity. The voltage divider circuit is designed to realize the terminal load self-calibration function. Experiments show that this sensor results in the characteristics of low reflection loss, good linearity, inline self-detection, and compatible with the GaAs MMIC process.
